# Impact of an Inpatient Palliative Care Consultation in Terminally Ill Cancer Patients

**DOI:** 10.7759/cureus.3016

**Published:** 2018-07-20

**Authors:** Brandon Blue, Radhakrishna Vegunta, Miriam Rodin, Setu Patolia, Nishant Poddar

**Affiliations:** 1 Hematology/Oncology, Saint Louis University School of Medicine, Saint Louis, USA; 2 Oncology, Roger Maris, Fargo, USA; 3 Geriatrics, Saint Louis University School of Medicine, Saint Louis, USA; 4 Pulmonary Critical Care Medicine, Saint Louis University School of Medicine, Saint Louis, USA; 5 Division of Hematology and Oncology, Saint Louis University School of Medicine, Saint Louis, USA

**Keywords:** cancer care, palliative care, terminal lung cancer

## Abstract

Abstract: Limited data are available to guide the timing of palliative care involvement in the treatment of cancer. We describe the referral patterns of inpatient palliative care consultations(IPCC) in advanced cancer patients in a tertiary care center.

Methods: A retrospective review was performed of IPCC for cancer patients from January 1, 2014, to December 31, 2014. Descriptive statistics are reported.

Results: IPCCs were requested for 245 cancer inpatients, of which 130 were male (53.1%) and 115 (46.9%) were female; 128 (52.2%) were Caucasian, 114 (46.5%) were African American, and 3 (1.2%) were another race. Of the 245 patients, 79 (32.2%) were newly diagnosed during the current admission, and the remaining 146 (67.8%) had been diagnosed previously. Fifty-seven (23.3%) patients were admitted to the intensive care unit (ICU) during hospitalization. Of the 39 patients (15.9%) who died during their hospital stay, 34 (87.0%) had an ICU stay during the hospitalization or died in the ICU. The most common malignancies were lung (71; 29.0%), pancreatic-biliary (33; 13.4%), lymphoma and leukemia (22; 8.9%), hepatocellular (18; 7.3%), head and neck (16; 6.5%), and upper gastrointestinal tract(GI) (16; 6.5%).

Conclusions: Our data show that 15.9% of terminally ill cancer patients with IPCC died in the hospital, the majority of whom died in the ICU. This was likely due to delays in the initiation of outpatient palliative care consultation, leading to an increased strain on tertiary referral centers. Our study highlights a racial disparity in the rate of IPCC in African Americans, compared to historical data.

## Introduction

While terminally ill cancer patients have complex medical and psychosocial needs at the end of life, there is no consistent standard to guide the appropriate timing for palliative care involvement in the medical treatment plan [1]. Indeed, a number of physicians view the early involvement of palliative care services as having a negative effect on a patient’s prognosis and expectations of treatment [2]. On the other hand, others advocate for early palliative care involvement, arguing that late involvement can lead to unnecessary hospitalizations and diminished quality of life [2]. In support of this argument, several studies have shown that the early implementation of palliative care can offer many benefits: improved quality of life, improved mood, greater overall survival, decreased costs of care, fewer admissions requiring high-level care, and fewer instances of in-hospital death [3-4].

While the latter data have helped to drive the earlier initiation of palliative care, in recent years, limited studies speak to the appropriate timing for the initiation of palliative care in the treatment of terminally ill cancer patients [5]. In clinical practice, clinicians must consider whether inpatient or outpatient palliative care consultations are better for patients. Despite studies that demonstrate the benefits of early palliative care involvement, research has shown that referral patterns among oncologists for patients requiring palliative services often occur late in the disease course [6]. To answer the question of whether inpatient or outpatient is superior, we must first understand referral patterns for palliative care in cancer patients. The purpose of this study is to describe referral patterns among inpatient palliative care consults for terminally ill cancer patients at a tertiary academic center.

## Materials and methods

A retrospective review was performed in which inpatient palliative consultations were extracted from an electronic health record databased for 245 adult cancer patients hospitalized between January 1, 2014, and December 31, 2014. Data were obtained for inpatient consultations for cancer patients that occurred in that year. We extracted the following data: demographics, diagnosis, floor/ICU status, and disposition. Data collection and analysis were reviewed for Health Insurance Portability and Accountability Act (HIPAA), human subjects, and financial concerns, and the study received institutional review board (IRB) approval. The statistical analysis was descriptive, including frequency distributions, means, ranges, and medians.

## Results

As noted, inpatient palliative care consults (IPCC) were obtained for 245 cancer patients, of whom 130 (53.1%) were male and 115 (46.9%) were female. One-hundred twenty-eight (52.2%) were Caucasian, 114 (46.5%) were African American, and three (1.2%) were of another race. The most common malignancies were lung (71; 28.9%), pancreatic-biliary (33; 13.4%), lymphoma and leukemia (22; 8.9%), hepatocellular carcinoma (18; 7.3%), head and neck (16; 6.5%), and upper gastrointestinal tract (GI) (16; 6.5%). Seventy-nine patients (32.2%) were newly diagnosed with cancer during the time of the initial IPCC. Fifty-seven (23.3%) patients were admitted to the intensive care unit (ICU) during hospitalization, of whom 11 (23.0%) had initial palliative care involvement of cancer patients during their ICU stay. Of the 39 patients (15.9%) who died in the hospital, 34 (87.0%) had an ICU stay during the hospitalization or died in the ICU. Of the 206 patients who survived hospitalization, disposition at discharge included home hospice (67; 32.5%), hospice in facility (27; 11.0%), home without hospice (71; 28.9%), and facility without hospice (39; 15.9%). In our cohort, 15.9% of admitted patients died in the hospital, with 87.0% of these deaths occurring in the ICU. Eighteen percent of the patients who died were receiving chemotherapy in the last 14 days of their life. For a breakdown of demographic data, please see Table 1.

**Table 1:** Demographics

Demographics	N = 245
Sex	
Male	130 (53.06%)
Female	115 (46.93%)
Age	
< 65 years	122 (49.79%)
65-75 years	71 (28.97%)
> 75 years	52 (21.22%)
Race	
Caucasian	128 (52.24%)
African American	114 (46.53%)
Other	3 (1.22%)
Admission status	
Floor	188 (76.73%)
Intensive Care Unit	57 (23.26%)

## Discussion

Our findings demonstrate a pattern of late initiation of palliative care in a large proportion of terminally ill cancer patients, as well as an increased use of high-level care among those patients who are introduced to palliative care in the hospital setting. These findings echo previous studies that have identified delays in the initiation of such services, despite evidence that the early initiation of palliative care can decrease the use of invasive procedures, the number and length of hospital stays, and in-hospital deaths. These results emphasize the need to improve time to initial palliative contact, especially for terminally ill patients with aggressive malignancies.

The rates of cancer patients dying in acute care hospitals varies. A Canadian study cites data that range from 39.0%-69.0%, depending on geographic location [7]. Our data show that 15.9% of terminally ill cancer patients with an IPCC referral died in the hospital. Of the latter group, 87.0% died in the ICU. This is likely due to delays in the initiation of palliative care consultation as outpatients, leading to increased strain on tertiary referral centers. A large national study showed 22.0% of eligible African American patients received IPCC in an urban academic medical center [8]. Our rate of IPCC in African Americans was 46.5%, higher than medical centers with a similar background being an urban academic medical center [8]. This highlights the disparity in access to palliative care services across various academic institutions. Currently, there is a known disparity and variation in the utilization of palliative care services among different races, with African Americans having the least expenditure of resources [9]. Given this data, we believe if African American patients received IPCC at higher rates, this would reduce the well-documented disparity in access to hospice care [10]. We believe if the African American community had expanded access to centers with outpatient palliative care services, the need for an IPCC would be reduced, leading to improvement in end-of-life care and overall quality of life.

While we note that this study shows outcomes from a single center, we believe these results will likely be reflected in most urban academic centers. Other limitations include our lack of long-term follow-up for patients after IPCC and a lack of readmission rates for those who chose various levels of supportive care after hospital discharge. While we did capture time to death, cause of death information would have strengthened this study and will be an area we plan to explore in future research.

## Conclusions

Future efforts should be made to promote early outpatient palliative care interventions to reduce ICU admissions, hospital re-admissions, and healthcare costs. Efforts also should be directed at establishing national standards to guide referrals to palliative services across healthcare institutions and especially academic institutions in light of the higher acuity levels managed by those hospitals. Given that we know early palliative care services improve end-of-life quality of care, efforts can be made to expand access to palliative care services in the African American community. Finally, we need to better understand the factors that lead to delays in outpatient palliative care involvement, from both the patient and provider standpoints.
